# The Molecular Genetic Interaction Between Circadian Rhythms and Susceptibility to Seizures and Epilepsy

**DOI:** 10.3389/fneur.2020.00520

**Published:** 2020-06-23

**Authors:** Christopher J. Re, Alexander I. Batterman, Jason R. Gerstner, Russell J. Buono, Thomas N. Ferraro

**Affiliations:** ^1^Department of Biomedical Sciences, Cooper Medical School of Rowan University, Camden, NJ, United States; ^2^Department of Biomedical Sciences, Elson S. Floyd College of Medicine, Washington State University, Spokane, WA, United States

**Keywords:** clock gene, neuronal excitability, voltage-gated sodium (Nav) channels, inward rectifying K channels, chronotherapy

## Abstract

Seizure patterns observed in patients with epilepsy suggest that circadian rhythms and sleep/wake mechanisms play some role in the disease. This review addresses key topics in the relationship between circadian rhythms and seizures in epilepsy. We present basic information on circadian biology, but focus on research studying the influence of both the time of day and the sleep/wake cycle as independent but related factors on the expression of seizures in epilepsy. We review studies investigating how seizures and epilepsy disrupt expression of core clock genes, and how disruption of clock mechanisms impacts seizures and the development of epilepsy. We focus on the overlap between mechanisms of circadian-associated changes in SCN neuronal excitability and mechanisms of epileptogenesis as a means of identifying key pathways and molecules that could represent new targets or strategies for epilepsy therapy. Finally, we review the concept of chronotherapy and provide a perspective regarding its application to patients with epilepsy based on their individual characteristics (i.e., being a “morning person” or a “night owl”). We conclude that better understanding of the relationship between circadian rhythms, neuronal excitability, and seizures will allow both the identification of new therapeutic targets for treating epilepsy as well as more effective treatment regimens using currently available pharmacological and non-pharmacological strategies.

## Introduction

Across evolution, nearly all eukaryotic organisms, including humans, have relied on predictable cycles of sustained activity and rest for survival. Cycles of activity and rest occur in essentially all cells of the body (peripheral oscillations), but they are largely governed by a primary oscillating mechanism (sometimes referred to as a “master clock”) that occurs in neurons of the suprachiasmatic nucleus (SCN) of the hypothalamus (1, 2). In addition to influencing the sleep/wake cycle, protein products of the primary oscillating mechanism have broad impact on homeostatic processes at the cellular (e.g., cell cycle regulation, energy metabolism) and systems (e.g., endocrine, immune, cardiovascular, pulmonary, gastrointestinal, and nervous) levels.

In the present paper, we review circadian influences relevant to nervous system dysfunction associated with epilepsy. We describe clinical studies and studies using experimental animals showing that alteration of genes mediating function of the mammalian biological clock can influence the expression of seizures, and conversely that seizures can alter the expression of clock genes and activity in related gene pathways. We then discuss the clinical significance of the relationship between the time of day and excitability of the nervous system leading to a seizure, highlighting studies that report patterns into which seizures cluster in several types of epilepsy.

An overarching postulate guiding this review is that circadian factors underlie mechanisms that lead to abnormal neuronal excitability in specific “epileptogenic” areas of the brain, resulting in seizures and epilepsy. At the same time, there is also focus on the idea that individual seizures disrupt both biological clock mechanisms and brain function related to circadian or diurnal rhythms. This may lead to biomolecular changes required to initiate the process of epileptogenesis, and establish chronic epilepsy. The goal of this work is to provide better understanding of the interrelationship between circadian rhythms, clock genes, and neuronal excitability, thereby providing deeper insight into the nature of seizure activity, and aiding initiatives to develop new strategies for the treatment of epilepsy.

## Time of Day and Sleep/Wake Effects on Seizures in Epilepsy

A seizure is defined broadly as a period of abnormal, synchronous excitation of a populous of neurons and neural pathways resulting from an imbalance of GABAergic and glutamatergic neurotransmission (3). Seizures may occur due to reversible insults such as infection, fever, trauma, hypoglycemia, stroke or tumor. They may be triggered by sleep deprivation or by withdrawal from drugs of abuse. In contrast, epilepsy is a chronic condition of unprovoked recurrent seizures, arising from a persistent dysfunction in neuronal excitability (4). When it develops as a result of an irreversible insult, it is known as acquired epilepsy. Epilepsy that develops in the absence of any known cause is generally considered to have a strong genetic component and is referred to as idiopathic. An epilepsy syndrome describes patients who can be grouped by their phenotypic similarities with respect to electroencephalography (EEG) findings, age of onset, response to medications and other clinical characteristics. The formal definition of epilepsy was recently updated by a Task Force of the International League Against Epilepsy (5).

For some people with epilepsy, seizures occur in a predictable pattern or cycle over the course of a month, week, or day (6). Seizures may be clearly associated with sleep/wake states. For other patients, seizures appear to be random events. Clinical studies making use of seizure tracking databases as well as those examining patients undergoing video-EEG monitoring have revealed significant trends between time of day and/or circadian phase and peak occurrence of seizures in specific types of epilepsy. Thus, analysis of records from the SeizureTracker database between the years of 2007 and 2015 (1,118 patients included) revealed that 14–22% of patients demonstrated seizure cycles >3 weeks, 7–21% showed strong weekly cycles, and over 80% exhibited a phenotype in which seizure rate was influenced either by the time of day (i.e., by the hour), circadian rhythms, or sleep/wake state (7). These data suggest that the majority of people with epilepsy exhibit seizures with some degree of periodicity.

Different seizure phenotypes have been shown to occur more frequently at different times of the day (we define daytime as 6:00–18:00 and nighttime as 19:00–5:00) and sleep/wake state. Focal seizures of temporal lobe origin have been documented to occur mainly during wakefulness and to peak in the afternoon and early morning (8–14). Seizures originating from the occipital lobe have also been linked to wakefulness and daytime (8, 9). In addition, wakefulness and daytime have been associated with generalized, atonic, myoclonic, and hypomotor seizures (15, 16). Wakefulness specifically has been tied to auras and epileptic spasms, as well as absence, gelastic and dialeptic seizures (9, 16, 17). Conversely, there are seizure phenotypes that typically occur during the night and while sleeping. These include automotor, hypermotor, tonic, and clonic seizures (9, 16, 17), along with focal seizures originating from the frontal (8–11) and parietal lobes (8, 16). Juvenile myoclonic epilepsy (JME), a common form of generalized epilepsy, is characterized by EEG and clinical seizures that are associated closely with the sleep/wake cycle, especially transition phases such as falling asleep and awakening (18).

In a few studies, the time at which certain seizure types peaked was influenced by patient age. For instance, in one report, focal seizures originating from the frontal lobe occurred typically at night in adolescents but during the day in infants, with a trend for increased seizure probability at night as age increased (15). An additional variable that warrants consideration is seizure severity, which appears to be highest during the day (19). In particular, status epilepticus has been reported to occur most frequently later in the day in infants and in the morning in older children (20, 21). For both infants and older children, status epilepticus occurrences appear to diminish at night.

Whereas seizures occur more often during wakefulness in patients with generalized epilepsy, interictal epileptiform discharges occur with greater frequency during sleep, predominantly non-rapid eye movement (NREM) sleep, with the highest rate of epileptiform discharges observed in the hour following onset of sleep and in the hour prior to the onset of wakefulness (22). Brief epileptiform electrical disturbances are not known to be directly correlated with behavioral seizures; however, individuals who exhibit abnormal EEG discharges are more likely to develop epilepsy (23) and drug treatment of interictal EEG abnormalities reduces epilepsy-associated comorbidities (24). Thus, it is possible that epileptiform discharges may represent a type of kindling phenomenon such that accumulation of electrical disturbances during sleep leads to the expression of seizures during wakefulness. Further research is required to fully elucidate the relationship between epileptiform discharges during sleep and behavioral seizures during wakefulness.

Interaction between sleep and seizures in certain forms of epilepsy occurs independent of the time of day, suggesting that there are fundamental mechanisms of sleep that increase the electrical excitability of neurons participating in seizure initiation. Sleep spindles are an electrographic signature of sleep that correlate in frequency with interictal spike-wave discharges (25) and may be biomarkers of epilepsy in humans (26). They are relevant in both generalized (27) as well as focal epilepsy (28) and display altered morphology in many epilepsy patients (29). Sleep spindles are also subject to regulation by circadian rhythms (30). They may occur independently or may occur in association with epileptiform discharges (31). In addition to serving as biomarkers of epilepsy, sleep spindles may also have functional significance (32), such as in absence epilepsy where they may evolve into spike wave discharges (33). The reticular nucleus of the thalamus controls both sleep spindles and epileptiform discharges (34), an anatomical correlation that is consistent with a functional role for sleep spindle mechanisms in seizures and epilepsy. Although animal models provide support for an association between sleep spindles and epilepsy (35), recent evidence indicates that spike-wave discharges are not pathological sleep spindles (36). Additional research is required to clarify possible mechanistic links between changes in sleep spindle characteristics and epileptogenesis.

Other sleep-related phenomena to consider in relation to epilepsy include neocortical slow oscillations, which are seen in cortical and thalamic neurons when consciousness is suppressed, including during coma and natural sleep (37). At the cellular level, these oscillations are characterized by depolarized “Up” states and hyperpolarized “Down” states, with neurons in the Up state having increased firing rate and decreased input resistance. A few studies suggest that these states are also present in epileptic seizures that occur both in humans (38) and rodents (39), which is interesting, given that many seizure types also inhibit consciousness. One study using *in vivo* whole-cell recordings in a rat model of focal limbic seizures found that the membrane potential of frontal cortical secondary motor cortex layer 5 neurons oscillated during seizures and produced Up and Down states comparable to those seen during anesthesia (39). Taken together, these findings shed light on mechanisms potentially linking sleep with certain types of seizures in epilepsy.

Within the context of sleep influences on epilepsy, syndromes of sleep-related hypermotor epilepsy are noteworthy (40). These syndromes include what previously were known as nocturnal epilepsies; however, a change in name was recommended to emphasize that these epilepsies are associated with sleep rather than the time of day. Sleep-related seizures involve characteristic and complex motor features that are often unique to individual patients (40). They generally arise from frontal cortex although they may also originate from other cortical areas. Compared to patients with seizure foci in the frontal cortex, sleep-related hypermotor seizure patients with foci in other cortical areas exhibit seizures with a longer duration and a shorter electrographic to behavioral latency (41).

There is a strong association between focal cortical dysplasia (FCD) and medication refractory sleep-related hypermotor epilepsy (42). FCD is classified into several types based on severity and cortical pathology, and is caused in part by somatic cell mutations that lead to abnormal development of the cerebral cortex (42). Mutations in genes involved in the mammalian target of rapamycin (mTOR) signaling cascade and the GATOR complex that negatively regulates mTOR signals have been implicated in FCD with sleep-related hypermotor epilepsy, including *DEPDC5, NPRL2*, and *NPRL3* (43).

**Figure 1:**
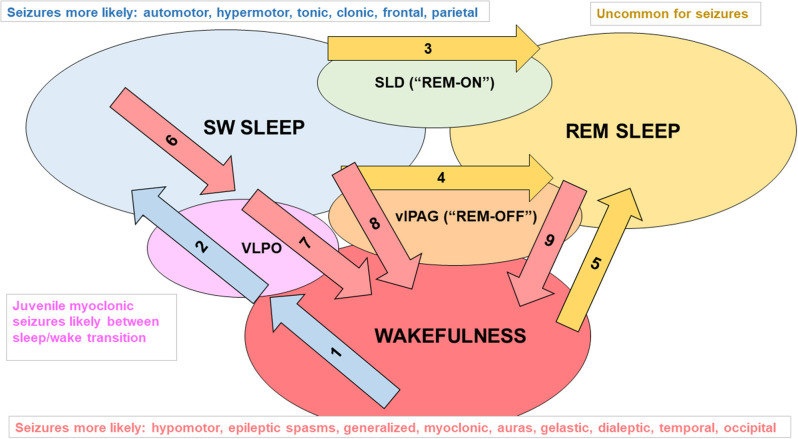
Examples of factors that promote changes in sleep/wake state and seizure types associated with each sleep/wake state. Mammals have two main cycles: the 24 h sleep/wake cycle and multiple 90 min slow wave sleep (SWS, i.e., NREM)/REM sleep cycles that typically occur within a 6 to 8 h period of sleep/inactivity. The sleep/wake state of an individual is influenced by various levels of endogenous clock products (that oscillate in a circadian rhythm) as well as external stimuli (zeitgebers) and allostatic factors (relate to the regulation and maintenance of sleep homeostasis) that act on the suprachiasmatic nucleus (SCN). Daylight is an example of a zeitgeber. It acts on melanopsin-containing retinal ganglion cells that transmit signals indicating the levels of environmental light (and thus the likely time of day) through the retinohypothalamic pathway to neurons of the SCN (49). Diminishing daylight reduces the inhibitory signaling of SCN projections to the pineal gland, enabling melatonin secretion. The SCN also regulates sustained sleep/wake states, as lesions of the SCN in rodents disrupt circadian rhythms and lead to random short bursts of sleep and wakefulness (50). *Influences on flux in the direction of SWS*: Arrow 1: adenosine accumulating from daytime glycogen use, increased release of melatonin in the evening and beyond; Arrow 2: adenosine activating ventrolateral preoptic area (VLPO) “sleep” neurons (foot on SWS gas pedal) (51–53), decreased activity of orexinergic neurons, and allostatic factors (e.g., leptin release after large dinner) promoting VLPO firing. *Influences on flux in the direction of REM sleep*: Arrow 3: glutamate activating the “REM-ON” neurons of the sublaterodorsal nucleus (SLD, foot on REM gas pedal) (54); Arrow 4: decreased excitatory input to REM-OFF neurons of the ventrolateral periaqueductal gray matter (vlPAG, foot off REM brakes); Arrow 5: orexinergic dysfunction (e.g., narcolepsy). *Influences on flux in the direction of wakefulness*: Arrow 6: adenosine levels diminishing from glycogen regeneration, decreasing melatonin secretion; Arrow 7: VLPO receiving inhibitory input from “wakefulness neurons” including orexinergic neurons in the lateral hypothalamus, noradrenergic (NE) neurons in the locus coeruleus, serotonergic (5HT) neurons in the raphe nuclei, histaminergic neurons of the tuberomammillary nucleus, and cholinergic neurons of the pons, basal forebrain, and medial septum (foot off wake brake), and a lack of excitatory adenosine (foot off SWS gas pedal); arrow 8: diminished firing of NE and 5HT at vlPAG (foot off wake brake); arrow 9: REM-OFF activated by orexinergic, 5HT, and NE neuronal firing (foot on wake gas).

Pharmacotherapy of sleep-related epilepsies is syndrome dependent (44). Progress in understanding the genetic contributions to sleep-related epilepsies (45), especially the role of mutations in genes that encode subunits of acetylcholine receptors (46), has led to more rational treatment strategies including nicotine patches and other drugs that modify cholinergic neurotransmission; however, like common forms of epilepsy, up to one-third of patients are refractory to available medications (47, 48). Despite advances in making diagnoses for the sleep-related epilepsies based on genetic testing, causative mutations are documented in only about 30% of cases (45). Figure 1 [based on (55)] summarizes the types of epilepsy in which seizures cluster in daily patterns, relating sleep/wake states with major factors that promote changes in circadian phase.

### Terminology and Definitions

#### Zeitgebers

Environmental stimuli (e.g., blue light or temperature) that are cues to entrain the circadian rhythm of an organism.

#### Diurnal Rhythm

The oscillation of a phenotype (e.g., feeding behavior or gene expression) over the course of a day in the presence of a zeitgeber.

#### Circadian Rhythm

A daily (roughly 24 h) behavioral or physiological process of an organism that is regulated by an endogenous, entrainable oscillator, which is maintained in the absence of zeitgebers.

#### Suprachiasmatic Nucleus (SCN)

A brain region within the hypothalamus, and dorsal to (i.e., above) the optic chiasm, that is responsible for controlling circadian rhythms.

#### Zeitgeber Times (ZTs)

ZTs relate to the current position of an organism within the light/dark cycle (56). In the context of preclinical research, laboratory animals are typically subjected to 12 h of light and 12 h of darkness. In this example, ZT0 represents light onset, whereas ZT12 represents darkness onset (i.e., lights off).

#### Circadian Times (CTs)

A standard unit of time based on the endogenous free-running period of a rhythm. For example, CT can relate to the current phase within the entire span of the circadian period that is being experienced by the organism in the absence of a zeitgeber.

#### Diurnal vs. Nocturnal

These two terms typically relate to the phase in the light/dark cycle in which the species or individual in question is active. Diurnal organisms such as humans are active and exhibit catabolic activity mostly during the light phase, whereas nocturnal organisms such as mice and rats are active and exhibit catabolic activity mostly during the dark phase. Consequently, diurnal and nocturnal organisms are in an inactive/anabolic state during the dark and light phases, respectively.

More information on terminology is found in Karatsoreos and Silver (56), Karatsoreos Eban-Rothschild and Bloch (57), and Voigt et al. (58).

## Core Clock Mechanisms Influence Neuronal Excitability

**Figure 2:**
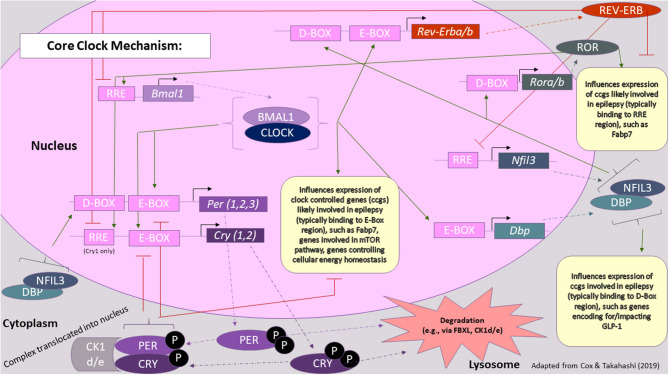
Core clock mechanism in the context of epilepsy. The core clock mechanism consists of three main feedback loops, which are thought to involve PER/CRY, REV-ERB/ROR, and DBP/NFIL3, respectively. Key proteins involved in these loops, whose levels fluctuate over the course of the day, are thought to also influence the expression of genes involved in seizure susceptibility or epileptic processes. Green arrows represent transcriptional activation, whereas red lines represent transcriptional repression. Rectangles represent genes and other DNA sequences of note, whereas ovals represent proteins. For a more comprehensive review on the processes underlying the molecular clock, please refer to (59) [Figure modified from (59)].

Time of day-dependent gene expression pathways generate circadian rhythms in most all somatic cells in the body, taking cues from the SCN and hypothalamus driven by zeitgebers (See Terminology Section). Critical protein products of this primary oscillating mechanism are encoded by period (*Per1, Per2, Per3*) and cryptochrome (*Cry1, Cry2*) genes (49). Figure 2 [adapted from (59)], provides a more comprehensive summary of core circadian clock genes and also draws connections between critical clock feedback loops and epilepsy. Overall, two main feedback loops lead to the cyclical fluctuation of levels of “clock proteins” that go on to activate/inactivate other biomolecular processes/substrates (e.g., “clock-controlled genes”) in a time-dependent manner, i.e., circadian regulation (60). These oscillations occur in both primary time-keeping cells (i.e., those of the SCN) and peripheral cells (i.e., essentially all other cells, both nervous and otherwise), though the circadian phase may be shifted from tissue to tissue within an organism, depending on how the tissue is connected to or responds to signals originating from the SCN, including photic and non-photic cues (i.e., feeding schedules).

Insight into the relationship between circadian rhythms and seizures comes first from studies showing that the time of day regulates neuronal excitability (61). Circadian-associated changes in intrinsic membrane properties of SCN neurons modulates inputs from other brain areas including thalamus and retina, especially at synapses utilizing GABA, a neurotransmitter whose altered function has been long-associated with epilepsy (62–65). GABA receptor activity governing excitability of SCN neurons changes in a circadian fashion via degradation mediated by a signaling mechanism involving the clock-related gene *Fbxl4* (66). Interestingly, mutations in *FBXL4* cause a mitochondrial disease characterized by a clinical syndrome that includes epilepsy (67).

Diverse ion channel proteins expressed uniquely in various subtypes of neurons determine the electrophysiological properties of neuronal membranes. In primary time-keeping neurons, cellular redox conditions govern circadian-dependent fluctuations in ion channel function and membrane excitability that underlie time of day-related behavior (68, 69). Specific potassium channels have emerged as particularly relevant to time of day effects on neuronal excitability. In a discovery approach to the elucidation of circadian pacemaker mechanisms in *Drosophila melanogaster, Ir*, a gene encoding a protein that mediates an inward-rectifying potassium current was identified as playing a key role in regulating the excitability of pacemaker neurons (70). Reducing *Ir* expression in pacemaker neurons increased larval light avoidance and lengthened the period of adult locomotor rhythms, consistent with increased excitability, whereas increased *Ir* expression eliminated daily behavioral rhythms and dampened PER protein oscillations. Mutations and common variants of *KCNJ10*, the gene encoding the inward-rectifying potassium channel Kir4.1, are associated with rare and common forms of epilepsy, respectively (71–74). Studies documenting the important role of Kir4.1 in the retina (75) and altered retinal function in epilepsy patients with *KCNJ10* mutations (76) suggest a potential mechanistic link between circadian rhythms and epilepsy that deserves further examination.

Melatonin is the primary hormone integrating circadian rhythms and sleep/wake cycles, a role that it fulfills by directly regulating neuronal excitability. Melatonin receptors signal through G protein-gated inwardly rectifying potassium (GIRK) channels in SCN neurons to regulate resting neuronal membrane potential via hyperpolarization (77). Melatonin receptor-linked GIRK channel activation in SCN neurons leads to phase advance (early sleep), and both GIRK2 protein expression and current amplitude oscillate over the sleep/wake cycle (77, 78). This suggests that the gene encoding GIRK2 (*KCNJ6*) is under clock control. Interestingly, GIRK2 deficient mice demonstrate increased susceptibility to chemically-induced seizures and even exhibit spontaneous generalized seizures (79). Conversely, activation of GIRK activity increases seizure latency and prevents convulsions in epileptic animals (80). GIRK channels have also been shown to be relevant to temporal lobe epilepsy (TLE). In one line of study, kainic acid-induced status epilepticus led to cleavage and dysfunction of GIRK1 and GIRK2 in rodent hippocampal tissue (81), further promoting hyperexcitability during the day, a period when individuals with TLE are most susceptible to having seizures (17). Thus, the relationship between GIRK and seizures may be bidirectional, both at the SCN and other neuronal populations.

The relationship between mechanisms of melatonin action and neuronal excitability related to seizures and epilepsy has received considerable attention. In animal models, the earliest studies show that melatonin inhibits expression of acute seizures induced by either GABA antagonists or glutamate agonists (82). These results are extended further by studies showing melatonin has anticonvulsant activity against hyperthermia-induced seizures and seizures induced by direct application of penicillin to the cerebral cortex (83, 84). It also decreases the frequency of spontaneous seizures arising after kainic acid-induced status epilepticus (85). Exogenous melatonin also has value as an adjunct to other anticonvulsants. Thus, it augments the anticonvulsant activity of phenobarbital in neonatal rats and of phenytoin and carbamazepine in mice (86, 87). Moreover, limbic epilepsy developing after status epilepticus induced by pilocarpine was significantly worse in rats that were pinealectomized, suggesting that melatonin deficiency facilitates epileptogenesis (88).

The anticonvulsant action of melatonin is mediated via specific melatonin receptors that control seizure threshold (89, 90). Further, the melatonin receptor agonist ramelteon possesses anticonvulsant effects and newer melatonin receptor drugs are under development (91–93). Clinical studies show that melatonin rhythm is normal in patients with epilepsy, but compared to control individuals, melatonin levels are low (94, 95). Thus, despite evidence that melatonin may be proconvulsant under some conditions, it is recognized as having potential therapeutic value in epilepsy and has been the focus of trials as an add-on therapy (96–98). In some cases, temporal clustering of seizures is in phase with the nocturnal rise in circulating melatonin, suggesting a potential difference between the physiologic role of endogenous melatonin and its action when administered as a drug (99). This idea is supported by a study in amygdala-kindled rats in which melatonin was effective at reducing seizure frequency even though the highest after-discharge threshold (i.e., lowest seizure susceptibility) was in the morning when melatonin reached trough levels (100).

Studies in *Drosophila* and mouse clock neurons led to discovery of a general mechanism for circadian control of membrane excitability. It involves two distinct sets of ion channel systems: a system of sodium channels that is upregulated in the morning and that mediates wakefulness, and a system of potassium channels that is upregulated in the evening and that mediates sleep (101). Functional upregulation of BK potassium channels to dampen excitability occurs by reduction of channel inactivation in the mouse SCN (102). Critical sodium currents are regulated by the activation of glycogen synthase kinase 3 (GSK3), which itself is activated via phosphorylation in a circadian-dependent fashion and contributes to neuronal excitability through regulation of the persistent sodium current, *INaP* (103, 104). *INaP* exhibits a day/night difference in peak magnitude, and inhibition of GSK3 in the SCN suppresses persistent sodium current and reduces spontaneous firing rate of these neurons during the light (inactive) phase in rodents (104). The findings suggest that GSK3 activation of persistent sodium current channels reduce action potential hyperpolarization, which in turn increases SCN firing rate during the light (inactive) phase. Interestingly, chronic GSK3 activation increases neuronal firing during the dark (active phase). GSK3 also regulates phosphorylation of the core clock gene protein product BMAL1, thereby providing an important feedback mechanism for circadian control (103, 105). Given that GSK3 inactivation is reported to increase during the active phase (104), it is possible that abnormal GSK3 activation could contribute to daytime seizures.

The hippocampus is a brain region involved in many common forms of epilepsy, especially TLE, and hippocampal neurons are subject to circadian control in ways that are similar to the SCN (106). Like SCN neurons, circadian changes in membrane properties of hippocampal neurons are associated with cellular redox mechanisms that regulate ion channel function and alter synaptic activity (107). All of the core clock genes are expressed in the various subregions of the hippocampus; however, like other brain regions, cyclic expression of clock genes in hippocampus may have a smaller amplitude compared to SCN and for some genes occurs in antiphase (i.e., 180 degrees out of phase) (108–115). Similar to the SCN, core clock proteins such as BMAL1 in the hippocampus are under the circadian control of GSK3 (116).

The role of clock genes in the hippocampus is not fully understood; however, several potentially important observations have been made. For example, hippocampal mechanisms related to long-term potentiation are under circadian control, and molecular mechanisms related to circadian control of synaptic plasticity as related to memory formation are highly conserved over the course of evolution (117, 118). Clock mechanisms in the brain, including hippocampus, involve Rev-Erbα (*NR1D1*), a nuclear receptor expressed in all examined hippocampal subfields which represses the transcription of circadian oscillators (119, 120). Also, in the dentate gyrus, *Per1* is linked to the process of neurogenesis (121). As mentioned previously, seizures experienced by patients with TLE tend to cluster during specific times of the day, suggesting a potential role for circadian-associated changes in hippocampal excitability (13).

Animal models support this concept. Analysis of slices prepared from rats killed at different points of the light/dark cycle reveal a larger steady-state amplitude of calcium current together with increased spike after-depolarization and slower adaptation of firing frequency after the start of the dark phase in CA3 neurons (122). Further, these changes were correlated with plasma levels of cortisol, a result consistent with a published clinical report showing higher cortisol levels are associated with greater seizure frequency in women with epilepsy being treated with anti-epileptic drugs (122, 123).

Although circadian effects on neuronal excitability of hippocampal neurons are well-documented, the specific events linking clock gene pathways to rhythmic changes in hippocampal synaptic activity, or to changes in the intrinsic properties of neuronal membranes, are not firmly established. *Kcna1* encodes the Kv1.1 voltage-gated potassium channel, which is highly expressed in pyramidal neurons of mouse hippocampus as well as in many other types of neurons. Mutant mice deficient in *Kcna1* exhibit a generalized seizure phenotype with diurnal periodicity and altered rest/activity rhythms (124). Electrophysiological study of hippocampal slices from epileptic *Kcna1* knockout mice reveals that the intrinsic passive properties of CA3 pyramidal cells are normal but antidromic action potentials are recruited at lower thresholds (125). In the same study, synaptically-mediated long-latency epileptiform burst discharges were triggered by mossy fiber stimulation. Furthermore, there is a circadian periodicity to spontaneous seizures, with frequency peaking in the first 12 h of the ZT cycle (125). Interestingly, more seizures arise out of sleep in diurnal conditions, whereas under constant darkness, the majority occur out of the wakeful state (126). Together, these results suggest that loss of Kv1.1 from CA3 neuronal axons and terminals results in increased recurrent axon collateral excitability and the seizure phenotype of *Kcna1* knockout mice (125). Also, of note, metabolism-based therapy with a ketogenic diet proved efficacious in lowering seizure frequency and restoring normal behavioral rhythms in this model (124).

A link between disrupted glial cell function and epilepsy has strong experimental support [reviewed in (127)]; therefore, circadian effects on glia may contribute to the timing of seizures in individual patients. Glial cells, including astrocytes, have been shown to regulate hippocampal processes, including synaptic transmission and excitability (128, 129), and to drive seizure threshold (130). TLE is often characterized by hippocampal sclerosis which includes synaptic reorganization, gliosis and neuronal loss (131). Astrocyte swelling associated with neuronal excitability is thought to occur through glial inwardly rectifying potassium channels (Kir channels) and water input via aquaporin-4 (AQP4) channels (132). Circadian and sleep/wake processes within astrocytes may also relate to time of day changes in excitability of hippocampal neurons. For example, diurnal changes in the expression and subcellular localization of brain fatty acid binding protein, Fabp7, occur in the hippocampus (133, 134). Fabp7 expression has been shown to increase in hippocampal astrocytes following kainic acid-induced seizures (135). Since Fabp7 expression is regulated by the circadian factors Rev-Erbα (136) and BMAL1 (137), and Rev-Erbα expression is affected by electroconvulsive seizure (138) while BMAL1 regulates seizure susceptibility (139), Fabp7 may represent a glial factor that contributes to epileptiform activity. Indeed, Fabp7 is known to control sleep/wake states in multiple species, including flies, mice, and humans (140, 141), therefore, Fabp7 expression in astrocytes may represent a functional node integrating neural activity, circadian rhythms and behavioral state.

Another neurotransmitter that links circadian rhythms and neuronal excitability is neuropeptide Y (NPY). This molecule is released by thalamic neurons, inducing phase advances in the SCN that are accompanied by suppression of *PER2* and are mediated by long-term depression of neuronal excitability in a phase-specific manner (142). This is consistent with findings in other brain regions where NPY-induced persistent hyperpolarization underlies mechanisms of energy homeostasis, anxiety-related behavior, and thalamocortical synchronous firing, the latter being particularly relevant to the pathophysiology of absence epilepsy (143). *PER2* expression is also dependent upon functional voltage-gated potassium channels including Kv4.2, which is encoded by *KCND2*, a gene that causes TLE when mutated (144, 145).

## Disruption of Core Clock Mechanisms: Relation to Seizures and Epilepsy

It is recognized that the interaction between circadian rhythms and epilepsy is bidirectional, with alteration of clock mechanisms acting as a susceptibility factor for epilepsy, and seizures acting as disruptors of the internal clock. Evidence from an experimental animal model of TLE that develops following electrically-induced status epilepticus reveals a phase shift in circadian control of population spike firing rate during the latent phase of epileptogenesis, demonstrating directly that circadian effects on neuronal excitability are relevant to the development of seizures and epilepsy (146).

Daily photoperiod controls excitability of the brain as reflected by behavioral responses to experimental seizure stimuli. In mice treated with pentylenetetrazol, an acute photoperiod change from 12h/12h to 18h/6h light/dark cycle lowered seizure threshold, whereas decreasing the length of the light phase had no effect. On the other hand, chronic photoperiod alteration, either shorter or longer, decreased seizure threshold compared to animals maintained under a 12h/12h light/dark cycle (147). Further evidence comes from a study showing that maximal electroshock seizure threshold is lowest during the light (inactive) phase of the light/dark cycle, a time-dependent variation that was absent in *Bmal1* knockout mice (139). *Bmal1* knockout mice also exhibit significantly lower seizure thresholds compared to wild type littermates, suggesting a direct link between molecular clock mechanisms and seizure susceptibility, and implicating BMAL1 as an influence on neuronal excitability leading to seizures (139). It is noteworthy that *Bmal1* knockout mice do not develop spontaneous seizures (i.e., epilepsy), suggesting that clock-related mechanisms function as susceptibility factors, providing a substrate upon which downstream factors interact to cause epilepsy. A corollary to this perspective is that clock-controlled genes influence intracellular signaling, membrane potential, and subsequent neuronal firing patterns, all of which are highly relevant to seizure manifestation and development of epilepsy.

In addition to controlling susceptibility to acute seizures, BMAL1 also has a role in the process of epileptogenesis, as suggested by a study showing that the time course of the reduction in hippocampal *Bmal1* expression in pilocarpine-treated rats parallels the development of spontaneous seizures (148). It is also likely that biomolecular mechanisms of epilepsy interacting with clock genes differ according to cellular subtype or brain region, given that different epilepsies are characterized by seizures that originate in distinct brain regions and occur at distinct points of the circadian cycle.

A possible means by which alteration in BMAL1 activity leads to seizures and epilepsy is via the mTOR pathway. Using a mouse model of tuberous sclerosis complex, a neurodevelopmental disorder involving epilepsy, in which the *Tsc1* gene was conditionally deleted in forebrain, BMAL1 activity was shown to be upregulated in an mTOR-dependent manner and reduction of BMAL1 to control levels was able to rescue the mutant phenotype (149). BMAL1 also acts to regulate translation of mTOR protein, linking activity of the mTOR pathway with circadian rhythms (150). The mTOR pathway has been implicated previously as being highly relevant to the development and treatment of epilepsy (151).

Regulation of cellular metabolism may be another way by which BMAL1 controls seizure threshold. Defects in cellular energy metabolism are known to be causative in certain forms of epilepsy and the anti-epileptic effect of the ketogenic diet may be related to alteration of cellular energy metabolism pathways (152). Disruption of BMAL1 causes hypoinsulinemia, diabetes, and defects in synaptic vesicle assembly, which could affect neuronal excitability and lead to seizures (153). A search for BMAL1 target genes revealed many that encode central regulators of metabolic processes, further supporting the possibility that regulation of seizure threshold by BMAL1 is related to changes in the expression of genes controlling cellular energy homeostasis (154). Interestingly, another circadian factor, the transcription factor D-albumin binding protein (DBP), affects hippocampal function and enhances seizure susceptibility via upregulation of glucagon-like peptide-1 receptor, a protein involved intimately in carbohydrate metabolism and cellular energy production (155, 156). Consistent with the findings on DBP/GLP-1 is evidence that glycogen in particular is an important energy molecule related directly to epilepsy (157). Relationships between BMAL1, seizure susceptibility and regulation of cell metabolism warrant further investigation with regards to the treatment of epilepsy.

A key protein interacting with BMAL1 is CLOCK, a molecule that is deficient in temporal lobe tissue from patients undergoing surgery for drug-refractory TLE (158). Deletion of the mouse *Clock* gene in cortical pyramidal neurons caused spontaneous epileptiform discharges in excitatory neurons associated with decreased inhibitory post-synaptic currents and decreased seizure threshold (158). The authors also report that the mutant mice have defects in dendritic spines similar to spine defects seen in human epileptogenic tissue. In a post-status epilepticus rat model, epileptogenesis is associated with loss of rhythmic expression of *Clock* transcripts and decreased levels of *Clock* transcripts at all ZTs studied, suggesting that *Clock* expression and neuronal activity in the hippocampus are linked (159). Subsequent study reveals that levels of *Clock* transcripts in hippocampus of pilocarpine-treated rats are not different from those in naïve rats at early time-points after treatment, but at later time points they are significantly reduced, suggesting that decreasing *Clock* expression is involved in the process of epileptogenesis (148). Dysregulation of *Clock* expression parallels disturbance of spontaneous locomotor activity, indicating a possible role for seizure as a non-photic behavioral cue, possibly related to neuronal activity in the hippocampus (148).

*Per1* is upregulated in the hippocampus following induction of experimental epilepsy, further evidence that seizures can perturb integral components of the clock (160). Clock mechanisms in extra-hippocampal brain regions are also perturbed by seizures. For example, one study showed that noise induces alteration of core clock gene expression in the inferior colliculus, a finding relevant to the elicitation of audiogenic seizures (161). Yet another study showed that electroconvulsive seizures in rats alter the expression of core clock genes in the frontal cortex (138). In the SCN, the time of day or ZT at which a seizure occurs has an influence on the rhythmicity of core clock gene expression and can also differentially affect sleep homeostasis (162). In the rat pilocarpine model, epileptic and naïve rats were compared at specific ZT points following the development of spontaneous seizures, with results showing that development of epilepsy is associated with alterations in rhythmic changes of all three period genes (*Per1, Per2*, and *Per3*) (159). In a subsequent study, *Per1* expression increased and *Per2* expression decreased at early time points after treatment, preceding the development of spontaneous seizures, whereas *Per3* expression was unchanged (148). Neuronal death in the hippocampus CA1 region in an experimental stroke model is enhanced in *Per1* knockout mice, a factor that may contribute to the risk for developing epilepsy following cerebrovascular insults (163). Overall, the strong bidirectional relationship between clock genes and seizures or epilepsy provides a compelling rationale for pursuing therapeutic strategies in this domain.

## Clock Genes as Causative or Risk Factors for Epilepsy

Mutations in human clock-related genes cause syndromes that include epilepsy in the phenotype. Specifically, mutations in the RORα gene (*RORA*) link to a syndrome known as Intellectual Developmental Disorder with or without Epilepsy or Cerebellar ataxia [IDDECA (164)]. Whole exome sequencing identified *RORA* mutations in eleven IDDECA patients exhibiting developmental delay and autism spectrum symptoms, with or without epilepsy or ataxia (164). A consortium organized by the International League Against Epilepsy (ILAE) is generating whole exome sequence data on 25,000 individuals with common forms of human epilepsy and a subset of patients with less common forms of developmental epileptic encephalopathy (DEE). Known as Epi25K (http://epi-25.org/), the project will also generate dense single nucleotide polymorphism maps for use in genome wide association studies (GWAS). The Epi25K group and ILAE consortium published preliminary findings on the GWAS and rare variants in the expanding cohort (165, 166). The first analysis of rare variants in over 9,000 patients from Epi25K is available on the web (https://epi25.broadinstitute.org/), and results show that rare mutations are found in the *RORA* gene significantly more often in the DDE patients than in controls. On the other hand, *RORA* mutations are not found in excess in patients with common forms of generalized and focal epilepsy, consistent with the rare IDDECA phenotype.

It is noteworthy in this context that the causative mutation in the “staggerer” (*sg*) mouse is a deletion in the *RORA* gene leading to defective Purkinje cell development in the cerebellum and an ataxic phenotype (167). *Sg* mice have spontaneous seizures, a finding consistent with the description of several rare human disorders in which development of epilepsy follows the development of ataxia, especially when the DNA polymerase gamma gene is mutated (168, 169). Thus, *RORA* gene mutations are linked to both ataxia and epilepsy in humans.

Similarly, mutations in the clock-related gene *FBXL3* link to two rare disorders. The first includes intellectual developmental disorder with short stature, facial anomalies and speech defects (IDDSFAS) (170), although these patients do not suffer from seizures. The second is a form of Batten disease known as Ceroid Lipofuscinosis, Neuronal, 5 (CLN5) involving a defect in lysosomal storage and degradation of damaged or unwanted proteins. Batten disease leads to intellectual disability, ataxia, visual disturbance, and epilepsy (171). This form of Batten disease is caused by mutations in the *CLN5* gene as well as mutations in *FBXL3*. Of interest is that these two genes overlap each other on chromosome 13 and transcribe from opposite DNA strands. Thus, it is possible that mutations found in *CNL5* or *FBXL3* could affect the expression or final function of either protein produced from this genomic region.

Mutations in *PER2* and *CRY1* result in advanced phase sleep disorder and delayed phase sleep disorder, respectively (172, 173); however, neither of these phenotypes includes epilepsy. Core clock genes including *CLOCK, BMAL1, FBXL21, PER1, PER3, CRY1*, and *NR1D1* do not show evidence of harboring mutations that cause epilepsy in humans. Likewise, there is little evidence to date from human studies that genetic variations within core clock genes act as susceptibility factors for epilepsy. This is most likely because there has not been a systematic investigation of polymorphisms in the core clock genes in large populations of homogeneous epilepsy patients with respect to the timing or clustering of seizures. Large scale GWAS studies, such as the one being performed by ILAE, include tens of thousands of patients; however, these studies suffer from patient heterogeneity, possibly masking true genetic associations that have a small effect on phenotype. Common variants of *CLOCK, PER2*, and *PER3* that had been associated with sleep disturbance were tested, but none showed association with JME in a cohort of <100 patients (174). Large scale GWAS with homogenous patient populations may reveal positive associations between core clock gene variations and epilepsy in the future, but for now, that hypothesis remains to be tested rigorously. Although there is yet little evidence that core clock gene variants predispose or cause epilepsy, *CLOCK* RNA and protein are downregulated in brain tissue resected from patients with TLE (158). It is unclear if the downregulation is due to promoter variation at the *CLOCK* gene itself or is a result of variation in other non-core circadian genes. It is also unclear whether this finding reflects a disease process or is due to the impact of AEDs.

The gene whose variation appears to have the largest impact on the development of epilepsy is *SCN1A*, encoding Nav1.1, the major voltage-gated sodium channel expressed in CNS neurons. Deleterious *SCN1A* mutations cause uncommon monogenic forms of epilepsy, and common variants increase the risk for more typical sporadic forms of epilepsy (166, 175). Of note is the fact that Nav1.1 is critical for the oscillatory function of the SCN, possibly contributing to sleep disturbance mechanisms in patients with common or even uncommon forms of epilepsy (176). Additional evidence that sleep disturbance in epilepsy is due to underlying alteration of circadian rhythm, rather than a side effect of anti-epileptic drugs, comes from studies of a mouse model of Dravet syndrome, a severe DDE, in which it was shown that an Nav1.1 mutation causing reduced interneuron excitability and seizures also causes sleep impairment (177).

## Chronotherapy in Epilepsy

Currently, regimens to treat a number of chronic medical conditions are beginning to apply concepts related to chronotherapy, including asthma (178, 179), hypertension (180), and type 2 diabetes (181, 182). The premise of chronotherapy is to administer drugs (or other interventions) at strategic points of patients' circadian rhythms, particularly time periods when the chronic condition is most severe or when the drug would exhibit optimal bioavailability or effectiveness (183). For instance, in type 2 diabetes, long and short-acting insulin preparations are administered strategically, such that predictable fluctuations in diurnal and meal-based glucose levels are adequately covered (182).

As mentioned above, the ability to predict the time period when it is most likely for an individual with a specific type of epilepsy to experience a seizure has opened up the possibility of utilizing chronotherapy to enhance the effectiveness of treatment for many people with epilepsy. The viability of chronotherapeutic strategies to optimize the efficacy of anti-epileptic drug (AED) treatment is supported by both clinical and basic research. In a study with rodents, the anti-convulsant efficacy of valproic acid varied as a function of the time in which it was administered (184). Though valproic acid increased the latency to the first pentylnetetrazol-induced seizure at all time points, the highest and lowest increases were observed when the drug was administered 7 and 19 h after light onset, respectively. The authors reasoned that these effects may have been influenced by circadian variation in drug disposition. For instance, some studies have found that drug clearance is greatest during dark exposure while others indicate that absorption of valproate is substantially delayed if taken after consuming a meal (14).

Results from human AED chronotherapy studies indicate direct clinical relevance. In one study, adults suffering from nighttime tonic-clonic seizures demonstrated improved seizure control and reduced side effects when a greater percentage of the daily divided dosage of phenytoin and carbamazepine was administered in the evening (185). By shifting the administration of AEDs from earlier in the day to 20:00, therapeutic drug levels were more readily achieved. This practice of uneven drug distribution is known as differential dosing. When children suffering from nighttime or early morning seizures were dosed differentially, such that the evening dose was twice that of the morning dose, at least a 50% reduction in seizure frequency was observed in 88% of patients (186). In another study with nighttime seizure patients, the differential dosing of clobazam (i.e., greater than half of the daily dosage was taken after 18:00) led to a median seizure reduction of 75% compared to a seizure reduction of 50% for the evenly dosed control group (187). Interestingly, the differentially dosed group also tolerated a higher total dose than the control group in terms of adverse effects.

**Figure 3:**
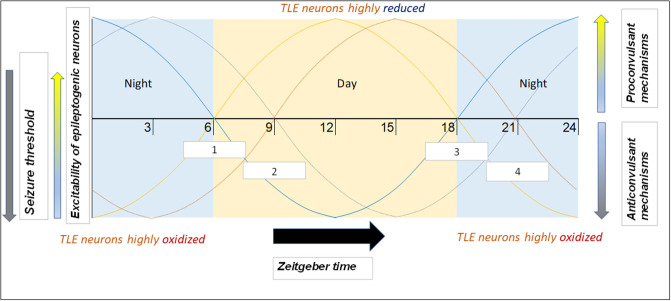
Example of how phenotype and chronotype permit implementation of chronotherapy for epilepsy, along with potential mechanisms responsible for time-based seizure susceptibility. This depiction portrays likely patient circadian (daily) seizure threshold/neuronal excitability patterns. Zeitgeber times are on the x-axis. Of the four “sample patients” illustrated, two have a morning chronotype (morning larks; curves depicted in lighter hues) and two have an evening chronotype (night owls; curves depicted in darker hues). Two suffer from focal seizures originating in the temporal lobe (orange hues) and seizures in the two others originate in the frontal lobe (blue hues). Since seizures are most likely to occur during the mid-day (12–15) and night (24–3) for temporal and frontal lobe epilepsies, respectively (9), therapeutic drugs may be best suited to be administered soon before the high seizure tendency time window (white boxes). Boxes 1 and 2 (corresponding to light orange and dark orange curves, respectively) represent AED time administration windows for TLE patients with morning and evening chronotypes, respectively. Boxes 3 and 4 (corresponding to light blue and dark blue curves, respectively) represent time windows for frontal lobe epilepsy (FLE) patients with morning and evening chronotypes, respectively.

An important consideration with regard to chronotherapy is patient chronotype (i.e., whether the individual is more morning or evening-oriented). Since people with evening chronotypes tend to go to bed later, their biological oscillating mechanisms (including those generating products that modulate seizure threshold/susceptibility) are likely shifted. That is, at 6:00 AM, the morning TLE patient will have different levels of relevant clock products than the evening TLE patient (as they are on different circadian times). For this reason, it may be reasonable to shift the recommended AED administration time according to the patient's chronotype and not base it on a specific time of day. Further, one study found that patients adapt self-administration of AEDs to their chronotype (188). Morning-oriented patients would often self-administer medications well before the recommended 8:00 time, whereas evening-oriented patients would often do so well after 8:00. The latter was especially true during “off days” (i.e., weekends, holidays), when the evening patients slept extra late in order to reduce their sleep debt. Therefore, an additional benefit of shifting AED dosing to later in the day is increased consistency of medication timing. This concept is illustrated in Figure 3.

In developing chronotherapeutic strategies in epilepsy, it is important to consider that seizures may influence chronotype. Thus, patients experiencing generalized seizures were 5 times more likely to have a late chronotype than healthy controls, and epilepsy patients do not appear to demonstrate the normal positive correlation between age and morning-proclivity (189). The former finding is interesting, given that the evening-heavy differential dose intervention employed by Thome-Souza et al. (187) was of most benefit to patients suffering from generalized seizures. However, genetic factors also influence chronotype, and so physicians must balance therapeutic regimens with promotion of healthy lifestyle decisions (e.g., preventing the sleep deprivation and circadian misalignment prevalent in evening-oriented individuals).

Applying chronotherapy to the management of epilepsy appears to be a prudent undertaking, particularly for patients with highly predictable seizure patterns or severe, drug-resistant epilepsy. Since every patient with epilepsy is unique, this practice would need to be patient specific. However, further research is required to better characterize the effects of chronotherapeutic approaches on all types of seizures and epileptic conditions. For instance, it may be useful to determine whether differential dosing favoring daytime AED administration enhances seizure suppression in patients that commonly experience focal seizures from the temporal lobe, since these seizures more frequently occur during the daytime and wakefulness. Interestingly, several recent studies suggest that certain biological mechanisms that change cellular redox state also correlate with neuronal excitability, and that many of these mechanisms occur in a circadian manner (68, 69). Indeed, diminished neuronal activity at night correlates with an electrochemically reduced intracellular state (69). Perhaps circadian variability in redox state may explain why seizures in TLE tend to occur during the day.

## Limitations

We accepted several limitations in the development of this review. One limitation was that our literature search did not include non-English language studies, and so it is possible that certain important information was omitted. Another limitation was that our literature search focused purely on peer-reviewed publications and did not consider information from dissertations, abstracts, or presentations at scientific conferences. Also, we did not include every relevant research study, but rather focused critical assessment on the most impactful work. Our literature review relied most heavily on the Pubmed database and we placed the greatest emphasis on studies published over the past decade. Our search strategy combined the terms “seizure,” “epilepsy” and “neuronal excitability” with the terms “circadian,” “diurnal,” “time of day,” and “sleep.” Overall, while our presentation encompasses the most relevant research conducted over the past decade, the approach we used to develop it did not fulfill journal criteria for a comprehensive systematic review.

## Summary

Despite the armamentarium of available treatments for epilepsy, many patients still experience variable numbers of seizures, some on a daily basis. Many factors influence the time of day at which a seizure will occur in a given individual with epilepsy, and some of these factors directly involve mechanisms that mediate circadian rhythms. Elucidation of genetic expression pathways underlying the oscillating function of SCN neurons, and the study of parallel pathways in epileptogenic regions of the brain (e.g., hippocampus, cerebral cortex), provides a basis for understanding links between circadian rhythms and seizures or epilepsy at the molecular level. In particular, the overlapping roles of specific ion channel proteins, both as determinants of SCN neuronal function and as susceptibility or causative factors for seizures and epilepsy, suggests potential approaches to the development of new anti-epilepsy treatment regimens. Mutations in certain ion channel genes important for SCN function, such as *SCN1A* and *KCNJ10*, are well-known to strongly associate with epilepsy phenotypes in humans, and have served for many years as leads for AED development without great success. However, a more complete understanding of how such genes interact with circadian factors could provide means to better exploit them as a source of druggable targets.

A critical link between circadian rhythms and expression of seizures relates to oscillations in the excitability of neuronal membranes. Cyclic expression of core clock genes in SCN neurons is synced with an oscillating pattern of membrane excitability, and testing whether similar core clock gene expression generates excitability oscillations in neurons from other brain regions will be important for determining their potential role in regulating circadian seizure susceptibility. For example, extra-SCN brain regions show molecular and cellular/neurophysiological rhythms that may be under autonomous control (190). Isolated hippocampal slice cultures from mPer2luc transgenic reporter mice show that PER2::LUC expression continues to persist over several circadian cycles (113), which could relate to circadian changes in hippocampal electrophysiology (117). In this way, vulnerable neurons in epileptogenic brain foci may be triggered to fire action potentials inappropriately, thereby initiating a seizure, at times when circadian-dependent membrane excitability is at a peak level. Whether SCN oscillations regulate circadian gene expression and electrophysiological properties of hippocampal or cortical neurons is poorly understood and remains an important area of future investigation. Deeper insight into the interaction between oscillatory neuronal activity in the SCN and the biophysical properties of epileptogenic neurons will lead potentially to the identification of novel AED targets and to the development of new medicines. In addition, development of detailed patient chronotypes, including comprehensive evaluation of the timing of seizure occurrence, may serve as a means to personalize therapy for people with epilepsy. This will not only facilitate the development of new drugs designed to target novel molecules identified at interface between circadian rhythms, neuronal excitability, and seizures, but will also lead to an increase in effectiveness of treatments that are currently available. Overall, further research into the relationship between circadian rhythms and epilepsy has the potential to lead to novel treatment strategies that could benefit many patients.

## Author Contributions

CR and AB performed the majority of the work including creation of figures. JG contributed oversight to the writing of sections Disruption of Core Clock Mechanisms: Relation to Seizures and Epilepsy, Clock Genes as Causative or Risk Factors for Epilepsy, Chronotherapy in Epilepsy, Limitations, and Summary. RB wrote the section Clock Genes As Causative or Risk Factors for Epilepsy and also contributed to other sections on the description of epilepsy and epilepsy seizure patterns. TF participated in the writing and editing of all sections and provided oversight to the entire project.

## Conflict of Interest

The authors declare that the research was conducted in the absence of any commercial or financial relationships that could be construed as a potential conflict of interest.
